# Patients Referred to a Norwegian Trauma Centre: effect of transfer distance on injury patterns, use of resources and outcomes

**DOI:** 10.1186/1752-2897-5-9

**Published:** 2011-06-16

**Authors:** Thomas Kristiansen, Hans M Lossius, Kjetil Søreide, Petter A Steen, Christine Gaarder, Pål A Næss

**Affiliations:** 1 Department of Research, Norwegian Air Ambulance Foundation, Drøbak, Norway; 2 Faculty of Medicine, University of Oslo, Oslo, Norway; 3 Department of Traumatology, Oslo University Hospital - Ullevål, Oslo, Norway; 4 Department of Surgical Sciences, University of Bergen, Bergen, Norway; 5 Department of Surgery, Stavanger University Hospital, Stavanger, Norway; 6 Prehospital Division, Oslo University Hospital - Ullevål, Oslo, Norway

**Keywords:** Injury, Trauma System, Interhospital Transfer, Norway

## Abstract

**Background:**

Triage and interhospital transfer are central to trauma systems. Few studies have addressed transferred trauma patients. This study investigated transfers of variable distances to OUH (Oslo University Hospital, Ullevål), one of the largest trauma centres in Europe.

**Methods:**

Patients included in the OUH trauma registry from 2001 to 2008 were included in the study. Demographic, injury, management and outcome data were abstracted. Patients were grouped according to transfer distance: ≤20 km, 21-100 km and > 100 km.

**Results:**

Of the 7.353 included patients, 5.803 were admitted directly, and 1.550 were transferred. The number of transfers per year increased, and there was no reduction in injury severity during the study period. Seventy-six per cent of the transferred patients were severely injured. With greater transfer distances, injury severity increased, and there were larger proportions of traffic injuries, polytrauma and hypotensive patients. With shorter distances, patients were older, and head injuries and injuries after falls were more common. The shorter transfers less often activated the trauma team: ≤20 km -34%; 21-100 km -51%; > 100 km -61%, compared to 92% of all directly admitted patients. The mortality for all transferred patients was 11%, but was unequally distributed according to transfer distance.

**Conclusion:**

This study shows heterogeneous characteristics and high injury severity among interhospital transfers. The rate of trauma team assessment was low and should be further examined. The mortality differences should be interpreted with caution as patients were in different phases of management. The descriptive characteristics outlined may be employed in the development of triage protocols and transfer guidelines.

## Background

The formalisation of trauma management has been associated with increased survival for injured patients, and trauma systems implementation is gaining momentum [1-5]. An important concept of trauma systems is to triage the most severely injured patients to a regional trauma centre, while patients not requiring this level of resources are managed at the nearest acute care hospital [6]. This necessitates effective prehospital triage and interhospital transfer; thus, these processes are key quality indicators of the trauma system [7,8].

Few European studies have addressed the population of transferred trauma patients. It has been reported that these patients are more often severely injured and in need of acute surgical or airway management than directly admitted patients [9-13].

Scandinavian trauma systems are in an immature state [14]. Consequently, pre- and interhospital transfer triage lacks specific guidelines. In some Scandinavian regions, the distance between the injury location and the nearest regional trauma centre may be great, and prehospital transport is often influenced by weather and topographical considerations [15,16]. This increases the role of local hospitals in resuscitating, stabilising and promptly transferring severely injured patients to higher levels of care [11]. The optimal time range for direct trauma centre admission vs. initial stabilisation at local hospitals is, however, yet to be determined.

Trauma patients transferred between hospitals are susceptible to inappropriate management at many levels of care; yet, this group of patients has received limited attention in trauma research. Consequently, we wanted to investigate the patterns of trauma transfer and describe the characteristics of patients transferred to one of the largest trauma centres in Northern Europe from hospitals within three regions of increasing transfer distance.

## Methods

### Trauma Services

Oslo University Hospital Ullevål (OUH) is the primary trauma centre for the one million inhabitants of Oslo and the surrounding municipality and is the referral trauma centre for the additional 1.7 million people in the mixed urban and rural parts of south-eastern Norway. The OUH is the only trauma hospital with neurosurgical services in this part of the country.

There were no formalised requirements for trauma competencies in the referring hospitals during the study period. However, increasing numbers of Norwegian hospitals have multidisciplinary trauma teams [17,18]. Great variation in the criteria for trauma team activation among hospitals has been reported [19]; the criteria for OUH were described previously [20]. In 2006, only four hospitals nationally had triage guidelines for interhospital transfer [18].

Paramedic-manned ground ambulances transport patients that are not transferred by helicopter emergency medical services (HEMS). Three HEMS bases with four helicopters are located in the southeast region of Norway; one is located within the trauma centre's primary catchment area. This HEMS base operates two helicopters, with one used mainly for interhospital transfers. There is also one military search and rescue helicopter base located in the region. All HEMS are staffed with certified anaesthesiologists or senior specialists-in-training. In Norway, only physicians perform prehospital endotracheal intubation on trauma patients.

### Patients and inclusion criteria

A trauma registry has been maintained at OUH since the year 2000, and demographic and clinical data on patients with moderate to severe injuries admitted to the trauma centre are prospectively recorded according to predetermined inclusion criteria: patients with Injury Severity Score (ISS) ≥ 9, torso and proximal penetrating injuries and any admissions activating the multidisciplinary trauma team [21]. Patients transferred > 24 hours after injury are not included in the registry unless the trauma team is activated. Patients admitted to OUH are admitted directly from the scene of injury, transferred from other hospitals or admitted via a primary health care casualty clinic located in central Oslo, 3 km from OUH. The casualty clinic manages minor trauma and is not staffed or equipped at hospital level. Patients initially managed at the casualty clinic were therefore not included in this study.

All patients from the trauma registry admitted to the trauma centre after transfer from other hospitals or directly admitted to the trauma centre between January 1, 2001 and December 31, 2008 were included.

### Definitions

#### Patient demographics and clinical characteristics

Injuries were coded according to the Abbreviated Injury Severity Scale (AIS) 1990 update 98 [22]. The ISS [23] and New Injury Severity Score (NISS) [24] were both included to allow comparison with other studies and optimal description of injury severity according to updated recommendations [25]. An injury was defined as penetrating if the injury with the highest AIS score was penetrating. The classifications "Head injury", "Spinal injury", "Thoracic injury" and "Abdominal/pelvic injury" included patients with AIS scores ≥ 3 in the respective anatomical region. "Polytrauma" included patients with AIS ≥ 3 injuries in two or more body regions based on the definition by Butcher et al. [26].

Injury mechanism categories included were "Transport", "Falls" and "Assaults". Not all injuries were assigned one of these three mechanisms; thus, the sums from each category do not correspond to the total.

The physiological variables were documented from the trauma centre emergency department records. To enable the inclusion of intubated and anaesthetised patients, prehospital data and data recorded at the transferring institution were used as described by Skaga et al. [27]. Where these data were missing, clinical notes were assessed and cases with altered consciousness were assigned a Glasgow coma scale score (GCS) of 8, while alert patients were assigned GCS of 15. Where no information was available, a GCS of 15 was assigned. Trauma team activation (TTA) described whether the multi-disciplinary trauma team was activated by a trauma call. The team may be activated prior to or during the patient's emergency department stay. Intubation described patients anaesthetised and endotracheally intubated prior to arriving at the emergency department. Transport by HEMS included patients who were secondarily transported by the HEMS services. The paediatric age group included patients aged 15 years or younger at the time of admission. The use of resources was described according to the Utstein definitions [25]. Mortality included deaths up to 30 days after injury.

#### Transfer distance

Transfers to OUH were grouped according to the distances by ground from the primary hospital. This was based on the *a-priori *hypothesis that the categories would yield groups of patients with dissimilar demographic and clinical characteristics. The distinctions were also regarded as purposeful in the interhospital transfer context. The hospitals were grouped in three separate distance intervals: 0-20, 21-100 and > 100 km. The 0-20 km group included hospitals within Oslo and the adjacent municipality, which is the primary catchment area of OUH for major trauma. The hospitals within the 21-100 km radius of OUH represented an uptake area in which primary admission to OUH by road transport may be an alternative to interhospital transfer [28], while this alternative may become less feasible for unstable patients in the > 100 km zone. The > 100 km category represented long-distance transfers, and the use of HEMS was expected to be higher within this group.

#### Study - time periods

To assess admission trends, some analyses were based on data in the first vs. the second half of the study period. This division was made arbitrarily to allow a detailed description of temporal changes by comparing two equally sized observational periods.

#### Study ethics

The study was approved by the Regional Ethics Committee (ref.no.2009/344)

#### Statistical Analysis

Mann Whitney U and Kruskal-Wallis-tests were used to compare two and more than two groups of patients, respectively. Results are presented as medians with inter-quartile ranges (*IQR*). *χ*^2 ^tests were used for categorical data. Mortality was compared using logistic regression and reported as an odds ratios (OR) with a 95% confidence interval (95% CI). Trends were assessed by simple linear regression with distance, age and the number of admissions per year as continuous values.

*Relative increase *refers to the increase in the median annual number of admissions in the second half of the study period relative to number of admissions in the first half.

Statistical Package for the Social Sciences, v.15.0 (SPSS, Inc., Chicago, IL) software was used for analysis and statistical significance was set at *p *< .05.

## Results

**Figure 1:**
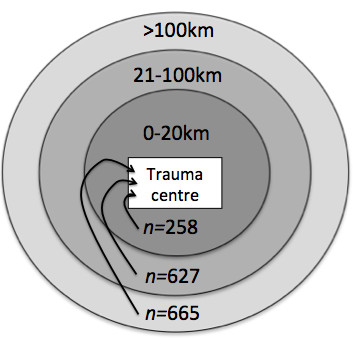
**Number of transfers according to distance from primary hospital to OUH during an eight-year period (2001-2008)**.

In total, 8.129 patients were included in the OUH trauma registry during the study period. For < 1% (*n=*78) of patients, data concerning the primary hospital were missing. In total, 698 patients were admitted from a primary health care casualty clinic and did not meet the inclusion criteria for the study, leaving 7.353 patients for further analysis. Of these, 5.803 were admitted directly from the scene of the accident, and 1.550 were transferred from 29 hospitals with a median transfer distance of 98 km (IQR: *51 km - 241 km*) (Figure 1).

The demographics and injury descriptions of all transferred patients vs. the directly admitted trauma patients are given in Table 1.

**Table 1:** Demographic and injury characteristics for 1550 patients transferred from other hospitals, and 5803 patients directly admitted, to OUH.

	Transfer	Direct	*p =*
Total *n*	*1550*	*5803*	

Age^#^	*39 *(21-60)	*33 *(22-48)	*< .001*

Paediatric *n *(%)	*192 *(12.4)	*669 *(11.5)	*.356*

Male *n *(%)	*1113 *(71.8)	*4159 *(71.7)	*.961*

ISS	*21 *(16-26)*^§^*	*10 *(4-19)*^§^*	*< .001*

NISS	*27 *(19-41)*^§^*	*11 *(4-27)*^§^*	*< .001*

SBP*	*127 *(110-145)*^§^*	*134 *(115-150)*^§^*	*< .001*

Hypotensive *n *(%)*	*94 *(6.6)	*301 *(5.4)	*.087*

GCS	*15 *(9-15)*^§^*	*15 *(13-15)*^§^*	*< .001*

GCS ≤8 *n *(%)	*374 *(24.1)	*850 *(14.6)	*< .001*

Penetrating Injury *n *(%)	*60 *(3.9)	*571 *(9.8)	*< .001*

Head Injury *n *(%)	*883 *(57.0)	*1366 *(23.5)	*< .001*

Spinal Injury *n *(%)	*360 *(23.2)	*471 (8.1)*	*< .001*

Thoracic Injury *n *(%)	*394 *(25.4)	*1190 *(20.5)	*< .001*

Abd/Pelvic Injury *n *(%)	*171 *(11.0)	*385 *(6.6)	*< .001*

Polytrauma *n *(%)	*492 *(31.7)	*1140 *(19.6)	*< .001*

Abbreviations denotes:

*ISS, Injury Severity Score; NISS, New Injury Severity Score; SBP, Systolic Blood Pressure; GCS, Glascow Coma Scale score*,

^# ^0+5 *cases had missing data on age. These were excluded from percentages and statistical analysis for age*.

*129+268 cases missing data for SBP. These were excluded from percentages and statistical analysis for SBP.

^§ ^median (IQR)

### Age

**Figure 2:**
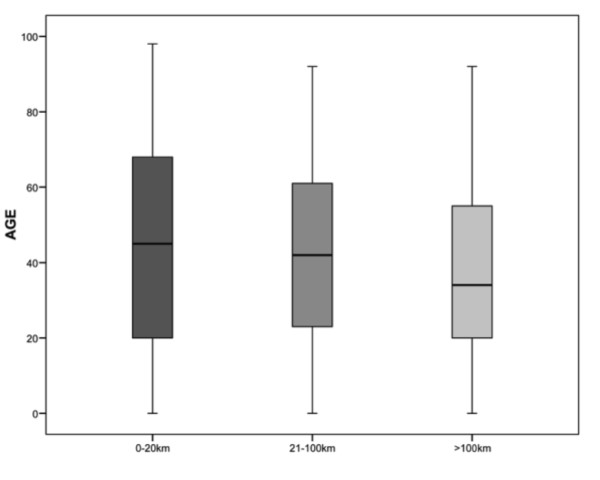
**Median age of transferred patients according to distance from primary hospital to OUH**.

A significant variation in age was found between the subgroups of hospital transfers (Figure 2) (*p *< .001, df: 2). Age was inversely related to transfer distance with a 3.7 (95% CI: 2.3-5.1) -year age decrease for each 100 km travelled (*p *for trend < .001). However, the proportion of patients in the paediatric age group was not significantly different among the different transfer groups (*p *= .134, df: 2). The median age of patients transferred after fall injuries was 25 years above the median for other transferred patients (56 (IQR: 30-71) vs. 31 (IQR: 19-48) *p *< .001).

### Mechanism of injury

**Figure 3:**
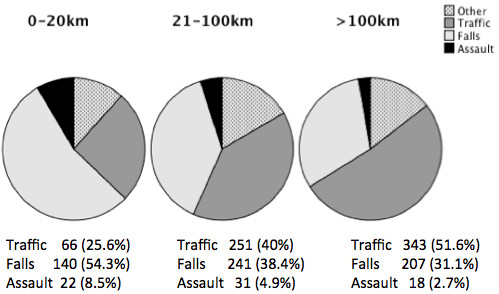
**Injury mechanisms, *n *(%), according to distances from pirmary hospital to OUH**.

The mechanism of injury varied according to the transfer distance (Figure 3).

Patients transferred from > 20 km were involved in transport accidents 1.8 times more often than those transferred from closer areas (*p *< .001). In contrary, assaults were 2.2 times more common within the 20 km radius (*p *= .001). Fall injuries were also significantly more common in patients from hospitals closer to the trauma centre (*p *< .001).

### Type of injury

Four per cent of the transferred patients sustained penetrating injuries, with no difference according to transfer distance (table 2). One in three patients transferred from hospitals > 20 km from OUH were polytraumatised. This was twice the proportion of polytrauma seen within the 20 km radius (*p *< .001).

**Table 2:** Injury severity, injury type and body region of injury in transferred trauma patients.

	Transfer Distance			
**Characteristics**	**< 20 km**	**21-100 km**	**> 100 km**	***p =***

Patients *n*	*258*	*627*	*665*	

Penetrating inj *n *(%)	*9 *(3.5)	*27 *(4.3)	*24 *(3.6)	.762

ISS	*17 *(14-25)*^§^*	*21 *(16-26)*^§^*	*21 *(16-29)*^§^*	< .001

NISS	*26 *(17-35)*^§^*	*29 *(21-41)*^§^*	*27 *(20-41)*^§^*	.004

Head inj *n *(%)	*174 *(67.4)	*360 *(57.4)	*349 *(52.5)	< .001

Spinal inj *n *(%)	*40 *(15.5)	*139 *(22.2)	*181 *(27.2)	.001

Thoracic inj *n *(%)	*31 *(12.0)	167 (26.6)	*196 *(29.5)	< .001

Abdominal/Pelvic inj *n *(%)	*12 *(4.7)	*83 *(13.2)	*76 *(11.4)	.001

Polytrauma *n *(%)	*44 *(17.1)	*212 *(33.8)	*236 *(35.5)	< .001

Abbreviations denotes:

*ISS, Injury Severity Score; NISS, New Injury Severity Score*.

*χ^2 ^-test performed with 2 degrees of freedom*.

^§ ^median (IQR)

More than two out of three transferred patients from hospitals within 20 km suffered from head injuries, a significantly higher proportion than patients from the more remote hospitals (*p *< .001). Injuries to other anatomical regions were more frequent, and injury severity scores were higher for patients in the two more remote groups (Table 2).

### Physiological parameters

Median systolic blood pressure decreased with increasing transfer distance (Table 3). The proportion of patients with a SBT < 90 mmHg was more than double in the two more remote groups combined vs. the ≤ 20 km group (*p *= .037).

**Table 3:** Vital parametres on arrival at trauma centre.

	Transfer Distance			
**Characteristics**	**< 20 km**	**21-100 km**	**> 100 km**	***p =***

Total *n*	*258*	*627*	*665*	

SBP *	*134.5 *(112-150)*^§^*	*129 *(110-145)*^§^*	*125 *(110-140)*^§^*	.001

SBP < 90 mmHg *n *(%)*	*7 *(2.7)	*43 *(6.9)	*44 *(6.6)	.076

GCS	*15 *(10-15)*^§^*	*15 *(9-15)*^§^*	*15 *(8-15)*^§^*	.476

GCS ≤ 8	*54 *(20.9)	*153 *(24.4)	*167 *(25.1)	.403

Abbreviations denotes:

*SBP, Systolic Blood Pressure; GCS, Glascow Coma Scale-score*.

*χ^2 ^-test performed with 2 degrees of freedom*.

* 36, 48 and 45 cases with missing data on SBP, respectively. These are excluded from percentage and statistical analysis for SBP and SBP < 90.

^§ ^median (IQR)

GCS scores were evenly distributed between the groups (Table 3). A higher proportion of patients from the more remote hospitals were endotracheally intubated before being transferred (Table 4), and if these were excluded, significantly more patients had GCS ≤ 8 among the ≤ 20 km referrals compared to the two more remote groups (9 of 177 vs. 3 of 638; *p *< .001).

**Table 4:** Resource use and outcome measures for transferred patients according to distance from trauma centre.

	Transfer Distance			
**Characteristics**	**< 20 km**	**21-100 km**	**> 100 km**	***p =***

Total *n*	*258*	*627*	*665*	

TTA *n *(%)	*88 *(34.1)	*318 *(50.7)	*402 *(60.5)	<.001

Intubation *n*(%)*	*51 *(19.8)	*252 *(40.3)	*312 *(47)	<.001

HEMS *n *(%)	*30 *(11.6)	*212 *(33.8)	*404 *(60.8)	<.001

ICU admission *n *(%)	*227 *(88)	*564 *(90)	*625 *(94)	.004

Days in ICU	2 (1-4)*^§^*	3 (2-8)*^§^*	4 (2-8)*^§^*	<.001

Days on Respirator	0 (0-2)*^§^*	0 (0-4)*^§^*	2 (0-5)*^§^*	<.001

Days in Trauma Centre	5 (3-9)*^§^*	6 (3-10)*^§^*	6 (4-11)*^§^*	.002

Mortality *n *(%)	*31 *(12)	*81 *(12.9)	*53 *(8)	.012

Abbreviations denotes:

*TTA, trauma team activation; HEMS, helicopter emergency medical system (patients transported by rotor-wing); *ICU, intensive care unit

*χ^2 ^-test performed with 2 degrees of freedom*.

* 0, 1 and 1 cases with missing data on intubation status. These are excluded from percentage and statistical analysis for SBP and Hypotension.

^§ ^median (IQR)

### Resources and Trends

In total, the transferred patients required 12.589 days of hospital stay, 8.959 days in the ICU and spent 5.397 days on mechanical ventilation during the eight-year period. The use of resources was significantly higher for those transferred from the more remote hospitals (Table 4).

**Figure 4:**
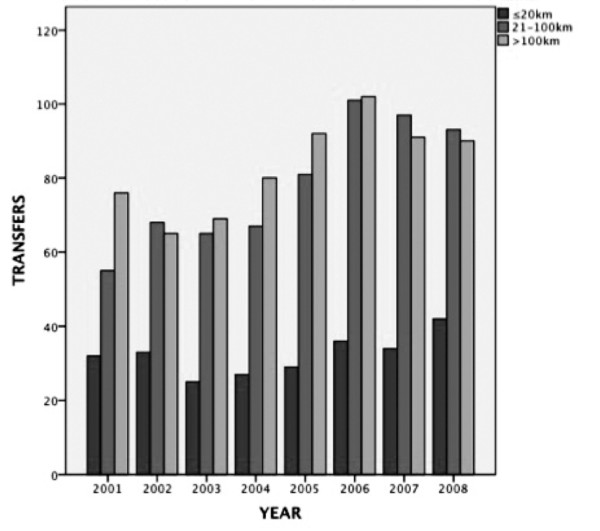
**Number of transfers per year by transfer distance to trauma centre from 2001 to 2008**.

The number of transfers increased by 11.7 per year (95% CI 5.8-17.6; *p *for trend = .003) (Figure 4), and there was a 36% relative increase in the median annual number of transferrals in the second half of the study period (*p *= 0.029). The 21-100 km group had the largest relative increase: 44% (*p *= 0.029). The increased number of admissions was not associated with a reduced injury severity. The median ISS and NISS tended to be higher for the second half of the study period (21 vs. 20 and 29 vs. 27), but this did not reach statistical significance (ISS *p *= .376; NISS *p *= .105).

### Management and Outcomes

The multidisciplinary trauma team was activated for 52% of all transferred patients (Table 4). The corresponding number for the directly admitted patients was 92% (5341 of 5803). When comparing the 21-100 km with the > 100 km groups, the latter were received by the trauma team significantly more often (*p *< .001) while there were no significant injury score differences (ISS: *p *= .732; NISS: *p *= .545).

Patients in the 0-20 km group were received by the trauma team the least often. For patients with severe injuries (ISS > 15), the trauma team was activated only half as frequently for transfers within 20 km (58 of 183 vs. 583 of 989) when compared to the other groups. Thus, the trauma team was not activated for more than two out of three severely injured patients within this group.

The proportion of patients managed by HEMS increased with increasing transfer distance (Table 4). These patients were also more severely injured compared to the non-HEMS transferred, with ISS 25 vs. 17 (*p *< .001) and NISS 34 vs. 27 (*p *< .001). Fifty nine per cent (378 of 645) of patients transferred by HEMS were intubated prior to arriving at the trauma centre vs. 26% of non-HEMS transfers, and 71% (460 of 646) were handed over to the trauma team vs. 39% of non-HEMS transfers.

Overall, the mortality of the transferred patients was 10.6% (Table 4), and it was lower in the > 100 km transfers than in the ≤ 100 km transfers (*p *= .003). The median age of patients that died in the ≤ 100 km group was 67.5 years (IQR: 45.25-79), which was 20 years older than the > 100 km transfers (47 years (IQR: 21-65)).

When assessing the mortality for patients transferred > 100 km vs. < 100 km with a logistic regression analysis adjusted for age and NISS, the mortality was lower in the > 100 km group than in the < 100 km group with an OR of 0.60 (95% CI 0.40-0.90 *p *= .014). The corresponding regression output when adjusting for age and ISS gives an OR of 0.57 (95% CI 0.39-0.83 *p *= .004).

## Discussion

A well-functioning interhospital transfer of patients is crucial for a trauma system, and the structure of the transfer process may serve as a quality indicator for regional trauma care [7,29]. We found an increasing trend for hospitals to refer injured patients to the trauma referral centre, indicating an informal process of trauma care centralisation during the study period. While polytrauma after traffic accidents were more common with longer transfer distances, the hospitals closer to OUH referred more elderly patients suffering head injuries after falls.

Three of four transferred patients in this study were severely injured (ISS > 15). However, the trauma team was summoned for only approximately half of all transfers and one third of the shorter transfers. This is in line with other Norwegian studies reporting less frequent trauma team activation for transferred patients [9,10,30]. Although there is no evidence that this practice led to suboptimal care in this study, the tendency to bypass the trauma team for transferred patients, especially from hospitals in the vicinity of the trauma centre, deserves further attention.

The outcome differences found in our study, with a higher mortality for ≤ 100 km transfers distances, may reflect the unequal phases of care for the different groups of patients when arriving at the trauma centre. As this study is based on data from the OUH trauma registry, the insufficient data capture on the time of injury is a limitation, and information on patients *not *transferred from the referring hospitals is needed to validly estimate the effect of transfer on the different groups. Regional or national trauma registries are needed to capture the complete transfer process [29]. There is probably also a *healthy enough for transfer *selection bias that is more pronounced in the peripheral regions. The reduction in average patient age with a longer transfer distance may also reflect such a selection of patients; as the transport distances increase, interhospital transfers after severe trauma may only be feasible for the younger and most resilient patients.

The importance of a systematic prehospital triage and effective transfer process has been highlighted in numerous studies [7,8,31,32]. Recent US field triage guidelines contain numerous criteria that directly triage patients to trauma centres [33], but for areas with long transport distances, the US recommendations are not specific [6,33,34]. Australian guidelines [28,35] allow up to 30 minutes excess transport time for direct triage to the trauma centre. By these standards, the long-distance transfers in our study (> 100 km) are in agreement with the recommendations, as a high proportion of patients were severely injured, and the transport time from the injury site to the trauma centre was more than 30 min. On the contrary, the closest transfers (≤ 20 km) to OUH are, by these definitions, initially undertriaged when brought to a non-trauma centre hospital. Studies have shown the importance of HEMS service and adequate advanced life support procedures for long-distance transfers [36,37]. For short-distance transfers, the triage protocols and interhospital transfer guidelines associated with trauma system implementation have been shown to reduce such undertriage and improve survival in other urban areas [8]. A Danish study showed more appropriate hospital admissions with the employment of prehospital physicians [38]. Protocols for direct triage to the trauma centre, bypassing local hospitals when the injury is suspected to be severe, are currently being implemented in the urban vicinity of OUH. This study indicates that such measures may allow a greater proportion of head-injured and elderly patients to receive earlier access to specialised trauma care.

The major challenge for developing triage protocols and transfer guidelines may lie in the group of intermediate transfer distances (21-100 km). According to trauma system recommendations [39], trauma centres in cooperation with referring hospitals should use available evidence and map available resources to develop written agreements to guide the flow of patients. The descriptive characteristics presented in this and future studies should form part of that evidence base.

## Conclusion

The interhospital transfer of patients to higher levels of care is a key process in trauma systems. We identified the characteristics of patients with short, intermediate and long transfer distances to a trauma referral centre, and we found heterogeneous demographic and clinical characteristics as a function of transfer distance. Prehospital triage protocols for areas close to the trauma centre and guidelines for the conduct of long-distance transfers are important components of trauma systems and are currently being developed in our region. Further studies are required to determine the optimal initial management of intermediate distance transfers.

## List of abbreviations

OUH: Oslo University Hospital; HEMS: Helicopter Emergency Medical Services; ISS: Injury Severity Score; AIS: Abbreviated Injury Severity Scale; NISS: New Injury Severity Score; GCS: Glasgow Coma Scale Score; TTA: Trauma Team Activation; OR: Odds Ratios; CI: Confidence Interval; SPSS: Statistical Package for the Social Sciences; IQR: Interquartile Range

## Competing interests

The authors declare that they have no competing interests.

## Authors' contributions

The corresponding author hereby states, on behalf of all authors, that all authors have contributed substantially to the work of this manuscript. All authors have contributed to the planning and conduct of the study, the interpretation of the results and the continuous revising of the article. TK and HML had the initial idea for the study. TK, PAN and HML have finalized the study design. TK has established the data file, conducted the analysis, reviewed the literature and drafted the initial manuscript. TK, KS and HML have drafted the final manuscript. All authors have contributed to, proof read and approved the final version of the manuscript.
